# The Montreal model: an integrative biomedical-psychedelic approach to ketamine for severe treatment-resistant depression

**DOI:** 10.3389/fpsyt.2023.1268832

**Published:** 2023-09-19

**Authors:** Nicolas Garel, Jessica Drury, Julien Thibault Lévesque, Nathalie Goyette, Alexandre Lehmann, Karl Looper, David Erritzoe, Shannon Dames, Gustavo Turecki, Soham Rej, Stephane Richard-Devantoy, Kyle T. Greenway

**Affiliations:** ^1^Department of Psychiatry, Faculty of Medicine, McGill University, Montréal, QC, Canada; ^2^Jewish General Hospital, Lady Davis Institute for Medical Research, Montreal, QC, Canada; ^3^McGill Group for Suicide Studies, Douglas Mental Health Research Institute, Montreal, QC, Canada; ^4^International Laboratory for Brain, Music and Sound Research, Montreal, QC, Canada; ^5^Department of Otolaryngology, Faculty of Medicine, McGill University, Montreal, QC, Canada; ^6^Division of Psychiatry, Department of Brain Sciences, Centres for Neuropsychopharmacology and Psychedelic Research, Imperial College London, London, United Kingdom; ^7^Health Sciences and Human Services, Vancouver Island University, Nanaimo, BC, Canada; ^8^Geri-PARTy Research Group, Jewish General Hospital, Montreal, QC, Canada

**Keywords:** ketamine, psychedelics, depression, pharmacology, psychotherapy, implementation science

## Abstract

**Background:**

Subanesthetic ketamine has accumulated meta-analytic evidence for rapid antidepressant effects in treatment-resistant depression (TRD), resulting in both excitement and debate. Many unanswered questions surround ketamine’s mechanisms of action and its integration into real-world psychiatric care, resulting in diverse utilizations that variously resemble electroconvulsive therapy, conventional antidepressants, or serotonergic psychedelics. There is thus an unmet need for clinical approaches to ketamine that are tailored to its unique therapeutic properties.

**Methods:**

This article presents the Montreal model, a comprehensive biopsychosocial approach to ketamine for severe TRD refined over 6 years in public healthcare settings. To contextualize its development, we review the evidence for ketamine as a biomedical and as a psychedelic treatment of depression, emphasizing each perspectives’ strengths, weaknesses, and distinct methods of utilization. We then describe the key clinical experiences and research findings that shaped the model’s various components, which are presented in detail.

**Results:**

The Montreal model, as implemented in a recent randomized clinical trial, aims to synergistically pair ketamine infusions with conventional and psychedelic biopsychosocial care. Ketamine is broadly conceptualized as a brief intervention that can produce windows of opportunity for enhanced psychiatric care, as well as powerful occasions for psychological growth. The model combines structured psychiatric care and concomitant psychotherapy with six ketamine infusions, administered with psychedelic-inspired nonpharmacological adjuncts including rolling preparative and integrative psychological support.

**Discussion:**

Our integrative model aims to bridge the biomedical-psychedelic divide to offer a feasible, flexible, and standardized approach to ketamine for TRD. Our learnings from developing and implementing this psychedelic-inspired model for severe, real-world patients in two academic hospitals may offer valuable insights for the ongoing roll-out of a range of psychedelic therapies. Further research is needed to assess the Montreal model’s effectiveness and hypothesized psychological mechanisms.

## 1. Introduction

Major depressive disorder (MDD) is highly prevalent and often devastating, afflicting more than 11% of Canadians across their lifetime (1). Approximately 30% of patients with MDD who receive evidence-based care will fail to respond to multiple conventional psychotherapeutic and pharmacological treatments (2, 3). Millions of individuals worldwide thus meet criteria for treatment-resistant depression (TRD), typically defined by a lack of meaningful improvement to at least two adequate trials of antidepressants (4).

TRD is associated with even greater societal costs and individual harms than MDD itself (5, 6). Patients with TRD have high rates of psychiatric comorbidities—anxiety disorders, sleep disorders, substance abuse, personality disorders, and intentional self-harm (7)—as well as greater functional impairment (6), elevated risks of repeated episodes (8), and increased long-term mortality overall and by suicide (9, 10). The complexity, chronicity, and severity of TRD therefore necessitates multidimensional psychiatric care (4).

Ketamine has emerged over the past few decades as one of the most promising new interventions for TRD (11), yet there is a great deal of debate about its subjective effects and uncertainty about how it may be optimally utilized. In this article we present the Montreal model, an approach to integrating ketamine into a broader biopsychosocial intervention for TRD that aims to deliver feasible and robust clinical benefits.

The model’s major components include a targeted psychiatric assessment, goal-oriented preparation, weekly concomitant psychotherapy, and a course of intravenous (IV) racemic ketamine infusions: six subanesthetic doses administered over 4 weeks administered in psychedelic-like treatment contexts. Its core objectives are as follows:

1. To utilize the rapid and powerful—yet typically transient—antidepressant effects of ketamine to facilitate evidence-based psychiatric care, including lifestyle modifications and medication optimization.
2. To administer ketamine with nonpharmacological psychedelic adjuncts, including music and psychological support, such that treatment experiences serve as opportunities for psychotherapeutic growth.
3. To enhance patient engagement in evidence-based psychotherapy by leveraging ketamine’s unique psychiatric benefits, including pro-cognitive and anti-suicide effects, and the psychological experiences of dosing sessions.

This model evolved over 6 years and more than 500 ketamine treatments administered to inpatients and outpatients with severe TRD at two McGill University hospitals (12). After being refined from these clinical experiences, the model was recently evaluated in a pragmatic RCT examining how music influences the physiological and psychological effects of ketamine (13). In the following sections, we overview the scientific literature and real-world “lessons learned” that informed the development of the Montreal model, which is subsequently detailed and discussed.

## 2. Methods

To provide the necessary context for the development of our model, we begin by reviewing the psychiatric management of TRD with a focus on ketamine, particularly its divergent “biomedical” and “psychedelic” bodies of literature and diverse care models.

### 2.1. Ketamine in TRD

#### 2.1.1. The need for new treatments

As the name would imply, the treatment of TRD is complex and challenging. Few specific treatments have direct evidence in unipolar TRD and only a handful have received specific regulatory approvals for this condition (defined in somewhat heterogenous ways): transcranial magnetic stimulation including the Stanford Accelerated Intelligent Neuromodulation Therapy, olanzapine/fluoxetine, and intranasal esketamine (14, 15). Many additional pharmacological agents for TRD are utilized in clinical practice and studied as off-label monotherapies and/or adjuncts, ranging from second-generation antipsychotics to lithium to thyroid hormones (16, 17).

The majority of real-world TRD patients end up receiving multiple psychotropic drugs (18, 19), often for years and with limited success. For instance, a recent 12-month cohort study of 411 TRD patients across multiple European countries reported high rates of polypharmacy and low rates of clinical response—only about 30% after 6–12 months of continual treatment (19). This study also found significant evidence of what might be called inertia or therapeutic nihilism: despite the cohort’s poor clinical outcomes, 60% of patients had no treatment changes after 12 months.

Apart from mainstay pharmacological therapies, electroconvulsive therapy (ECT) is arguably the most evidence-based treatment option for TRD (17). Though highly effective, ECT is associated with stigma, inconsistent availability, and significant side effects (largely due to treatments requiring general anesthesia) like amnesia (17, 20). These side-effects can interfere with concomitant psychotherapy, an adjunctive treatment with some evidence in TRD. A 2018 Cochrane review of psychotherapy for TRD, including six trials of 698 participants, found high rates of acceptability though only modest improvements with various approaches including Cognitive Behavioral Therapy (CBT) (21).

In this landscape, the rapid and potent antidepressant effects of subanesthetic IV racemic ketamine, a unique N-methyl-D-aspartate (NMDA) antagonist, have understandably generated significant excitement (22). On meta-analysis, approximately half of TRD patients will respond (>50% improvement in symptoms of depressions) within hours or days of a single 40-min IV ketamine infusion (23, 24). These results have prompted experts to describe subanesthetic ketamine as one of the psychiatry’s biggest pharmacological breakthroughs in decades (22).

While impressive, ketamine’s benefits typically fade within days or weeks (25). An extensive search for effective strategies to augment and/or maintain response has largely yielded disappointing results for a diverse variety of drugs, such as clonidine, D-cycloserine, lamotrigine, lithium, rapamycin, and riluzole (26). As a consequence, the most common strategy for prolonging ketamine’s benefits is simply to readminister ketamine (27, 28), despite a relative dearth of evidence for this practice’s long-term safety, efficacy, and feasibility (29).

The efficacy of ketamine must also be balanced against important safety considerations, including stimulatory hemodynamic effects (30), abuse potential (31) and long-term risks (at least in recreational ketamine users) like cognitive and bladder harms (32, 33). Indeed, ketamine abuse has resulted in serious urinary track complications and long-lasting adverse impacts on the nervous system and cognitive functioning, including loss of gray matter volume. These harms may only manifest with large quantities (up to 100 times greater than those used in clinical contexts) and neither ketamine or esketamine have been associated with iatrogenic dependence, cognitive decline (beyond transient memory disruption) or toxic bladder effects.

Outside of potential chronic adverse effect, ketamine can engender potentially distressing psychoactive effects in both recreational and medical contexts. These ketamine-induced altered states of consciousness have proven to be highly contentious, and have generated active debate regarding their therapeutic importance (25). Reflecting this debate, two relatively isolated bodies of research on ketamine in psychiatry have emerged, which we refer to as “biomedical” and “psychedelic.”

#### 2.1.2. Ketamine as a biomedical treatment of depression

In the dominant biomedical view, ketamine’s dose-related psychoactive effects are generally considered to be important for tolerability but are unrelated to efficacy (22, 25, 34, 35). This perspective instead emphasizes ketamine’s pharmacological and neural effects as the main mechanisms of therapeutic action (36). Given the perception that they are adverse events, ketamine’s psychoactive effects are typically mitigated and minimized using strategies like dose-minimization, reassurance/distraction, and potentially the administration of sedatives (34).

The origins of the biomedical approach to ketamine can be traced to a ground-breaking RCT, published in the year 2000, in which seven depressed patients received an intravenous dose of 0.5 mg per kg of bodyweight, infused over 40 min (37). This study marked a major turning point in the use of ketamine in psychiatry, building upon decades of clinical research of NMDA antagonists as experimental (psychotomimetic) models of schizophrenia and fundamental studies suggesting potential antidepressant effects (38). To this day, its dosing protocol remains the most commonly used around the world [though modifications have been found to be beneficial for particular patient populations (39) or to mitigate ketamine’s side effects (40)].

In the decades since, multiple clinical trials and larger observational studies have confirmed rapid antidepressant effects of intravenous ketamine in unipolar depression and, albeit with less evidence, bipolar depression (25, 41). A notable strength of this literature is the frequent inclusion of real-world TRD patients who rarely meet eligibility criteria for clinical trials due to their severity and comorbidity (42). Another uncommon strength of ketamine clinical trials is the frequent employment of active comparators like midazolam to reduce placebo effects and unblinding arising from psychoactive effects (43)—a rigorous methodological approach that may be broadly underutilized in depression research (44). In addition to strong evidence for antidepressant effects in complex real-world patients, ketamine can also produce benefits against suicidality (45, 46) and the cognitive symptoms of depression (47, 48), which are at least partially independent of core mood improvements (49).

As with other psychiatric treatments, the exact biological mechanisms underlying ketamine’s psychiatric benefits are subject to ongoing research and debate [as described in multiple recent reviews (50–52)], including the role of NMDA receptors in its antidepressive effects (53). Amongst many identified potential mechanisms, cellular and animal studies have demonstrated that ketamine can promote neuroplasticity (e.g., dendritic spinogenesis) and thereby reduce or reverse stress-related changes (54, 55), reduce inflammation (56), and dramatically alter neural dynamics (57, 58). Studies in humans have also found ketamine to transiently alter cerebral GABA and glutamate concentrations (59, 60), increase neural activity in areas associated with reward processing (61), and potentially normalize default mode network connectivity abnormalities of depression (62).

The promising clinical and preclinical ketamine research has also fueled a search for NMDA modulators that may convey ketamine-like antidepressant effects without its psychoactive effects. These efforts, however, have largely resulted in disappointment—existing drugs such as memantine have not proven to be effective and many drug candidates have been abandoned due to poor results of costly phase 1–3 clinical trials (63, 64). The only partial exception to this trend is intranasal esketamine, the s-enantiomer of ketamine, dosed such that its psychoactive effects are relatively minor (65). As mentioned above, esketamine has recently received regulatory approval in multiple countries for unipolar TRD in combination with conventional oral antidepressants (14).

Despite these approvals, the real-world uptake of intranasal esketamine has been limited. For instance, the Canadian Agency for Drugs and Technologies in Health and the United Kingdom’s National Institute for Health and Care Excellence (NICE) both recommended against public-payer esketamine adoption (66, 67). In their report, the NICE committee cited a lack of evidence for esketamine’s efficacy beyond standard biological or psychological therapies, uncertainty regarding the duration and sustainability of its benefits, and its relatively high costs.

These same limitations also apply to some extent to the current evidence-base for racemic ketamine, with the notable difference that its drug costs are significantly lower—an advantage that is somewhat offset by the greater costs associated with IV administration (68). Nevertheless, the tremendous need for novel treatments of TRD in conjunction with meta-analytic evidence of antidepressant effects for both IV racemic ketamine and intranasal esketamine have resulted in both finding active use in a variety of models of care (25, 69).

#### 2.1.3. Biomedical ketamine models of care

Typical biomedical models of care for parenteral ketamine most closely resemble ECT (17); i.e., ketamine treatments are administered twice or thrice weekly for several weeks (typically alongside oral medications) to induce remission, followed by further treatments at a decreasing frequency to maintain response (29, 65). The similarity between IV ketamine and ECT protocols is evidenced by at least two large, multicenter, head-to-head RCTs directly comparing them for TRD (70, 71). For instance, the Canadian biomarker integration network in depression (CAN-BIND) study protocol describes an ECT-like approach to IV ketamine involving acute treatment periods of thrice-weekly doses followed by maintenance doses decreasing in frequency across 6 months, to weekly and then to monthly (71). Esketamine is also utilized in a similar protocols featuring gradually less-frequent doses (65).

In addition to these ECT-like care models, there is also increasing interest in utilizing ketamine in ways more resemblant of oral antidepressants. For example, studies have evaluated at-home sublingual or oral ketamine doses prescribed up to three times per day (72, 73). Such approaches are significantly less studied than parenteral ketamine or intranasal esketamine (which also requires in-person administration due to the US Risk Evaluation and Mitigation Strategy) (74). However, and in spite of their increased potential for iatrogenic abuse, their significant feasibility advantages have resulted in substantial clinical growth— partially accelerated by relaxed telehealth prescribing rules in the US during the COVID-19 pandemic (74). Of note, some of the at-home ketamine care models might also be described as partially “psychedelic” given how they frame ketamine’s effects and incorporate, for example, telehealth psychological support (75).

All of these diverse approaches to ketamine, like the conventional treatments they resemble, have been associated with both significant benefits and drawbacks. Since antidepressant response is primarily achieved and maintained by repeated dosing—possibly on an indefinite basis—there is significant potential for long-term expense, inconvenience, imperfect adherence, and adverse side-effects (76, 77). Further, as experts have recently commented (78), biological models for TRD that do not explicitly address sociopsychological factors can paradoxically reinforce symptoms of depression like demoralization, passivity, and guilt. Lastly, ketamine’s acute psychoactive effects may cause distress—ranging from mild discomfort to acute panic attacks leading to premature treatment termination (79).

#### 2.1.4. Ketamine as a psychedelic treatment of depression

The important limitations to biomedical models of ketamine have contributed to rapidly rising interest in “psychedelic” approaches, which frame ketamine’s psychoactive effects in an extremely different light. Namely, drug experiences are not seen as treatment side-effects, but rather as essential drivers of potential benefits (80). While some experts reserve the psychedelic label specifically for agonists of serotonin 2A receptors like psilocybin (81), psychedelic treatment models have been utilized with diverse psychoactive drugs including MDMA (82), ibogaine (83), and cannabis (84), in addition to ketamine.

Psychedelic ketamine models have rarely been studied in modern clinical trials or implemented in real-world psychiatric services (85, 86). Nonetheless, they are far from new—scientific publications describing ketamine’s employment in psychedelic-like models appeared within a decade of its 1962 discovery (87). Their origins can be traced even further back, to traditional uses of psychedelic mushrooms and plants over many centuries (88), as well as to psychedelic-assisted psychotherapies that were first formalized in the 1950s (89).

There are many ways by which psychedelic and biomedical approaches differ. The connotations of the word *psychedelic* itself—“mind manifesting”—differ greatly from those of the pathological labels dissociative and psychotomimetic. Psychedelic approaches also utilize different dosing regimens, typically administering fewer treatments at higher doses with the explicit aim of engendering powerful alterations of consciousness (87), which are then shaped with treatment adjuncts like music and psychological support (90–93). In contrast to this strong emphasis on treatment contexts in psychedelic studies, most biomedical ketamine studies neither standardize nor even report non-pharmacological factors like the presence or absence of music during treatment sessions (94).

Unlike psilocybin, the vast majority of psychiatric ketamine studies fall into the biomedical rather than the psychedelic camp—a surprising divergence when one considers these drugs’ many similarities. Ketamine and psilocybin can both produce rapid improvements in psychiatric conditions like depression that persist for days or weeks beyond the excretion of the drugs and their metabolites (25, 91). Both ketamine and psilocybin appear to act as “psychoplastogens”: agents that rapidly boost neuroplasticity (95, 96). Moreover, recent neuroimaging research has revealed shared neural features of their effects despite pharmacological differences, including heighted neural signal diversity (97), as well as reduced within-network connectivity and enhanced between-network connectivity (98).

Perhaps most importantly, serotonergic psychedelic and ketamine experiences have significant phenomenological overlap. The altered states of consciousness induced by ketamine and psilocybin have both been long employed as experimental models of psychosis (87, 99, 100). When compared on psychometrically-valid scales administered in controlled experiments, like the Altered States of Consciousness Rating Scale (101), ketamine and psilocybin produce remarkably similar results, including significant elevations on subscales reflecting key hallmarks of psychedelic experiences like “insightfulness,” “audio-visual synesthesia,” and “changed meaning of precepts” (102). In fact, ketamine’s subjective effects are mostly distinguished from psilocybin by elevated scores on the “disembodiment” subscale (potentially reflecting its “dissociative” label).

The discrepancies between the dominant models of care for psilocybin and ketamine may thus owe more to their sociohistorical differences—including ketamine’s widespread use in medicine as a dissociative anesthetic versus psilocybin’s disappearance and recent re-emergence (103)—than to their pharmacological, neural, or phenomenological differences. Nevertheless, in depression, only psilocybin currently has strong evidence supporting the central tenet of psychedelics: that certain drug experiences like “emotional breakthroughs” and mystical experiences can be therapeutic (104, 105). While even those claims have been challenged by methodological criticisms and emerging data (106, 107), psychedelic-assisted psychotherapy models for psilocybin are undeniably more formalized and better supported by clinical research than equivalent models for ketamine.

According to a recent review, five small trials have studied ketamine-psychotherapy combinations, none of which could be described as psychedelic in their therapeutic approach and only one of which was in TRD (85). That trial evaluated whether CBT could prolong the benefits of ketamine in patients who responded to a course of six infusions over 3 weeks (108), yielding mixed results. Given that participants of this trial began CBT only *after* completing their series of ketamine infusions, it appears that no published trial has explored ketamine with concomitant psychotherapy to treat depression. Further, the only two other recent ketamine-psychotherapy trials both administered ketamine *disjointly* with (mindfulness-based) psychotherapy—that is, patients received ketamine during a course of psychotherapy but did not necessarily receive psychological support or psychedelic-like contexts *during* their ketamine infusions (109, 110).

#### 2.1.5. Psychedelic models of care

Given the lack of clinical trials employing psychedelic models for ketamine [despite encouraging retrospective reports (111–113)], we provide a concise review of the prevailing psilocybin-assisted psychotherapy (PAP) protocols which serve as the basis for most contemporary psychedelic ketamine models (112). We focus on the most common and distinctive approach involving one or two high doses of psilocybin, rather than the less common “psycholytic” approach—smaller doses used in conjunction with relatively frequent psychotherapy sessions (114)—or microdosing, the regular use of subperceptual doses of psychedelics without psychotherapy in a manner resemblant of oral antidepressants (115).

PAP protocols broadly aim to produce therapeutic psilocybin experiences in three distinct phases: preparation, treatment, and integration (90). The preparation phase of PAP entails patients and clinicians meeting for several hours in order to, for instance, exchange information, establish rapport, and set therapeutic intentions for the drug experiences (116). In some cases, this may entail defined preparatory practices such as mindfulness exercises (117), but most psychedelic studies do not detail specific activities.

During the second phase, referred to as treatment or dosing, patients undergo approximately 6-h psilocybin experiences, once or twice (91, 92), while receiving psychological support from their clinicians in comfortable and de-medicalized environments. The clinicians are often [but not always (118)] described as guides or “sitters” as opposed to therapists, partly to emphasize their non-directive therapeutic stance (90). Psychedelic experiences and associated insights are typically understood to arise spontaneously from patients’ minds rather than resulting from specific psychotherapeutic interventions (80), though nonpharmacological adjuncts like music are understood to be critical influences (94). Accordingly, one of the guiding precepts of this phase is “set and setting”; i.e., that psychedelic experiences are largely dictated by a patient’s mindset and the treatment setting (119), the influences of which are amplified by the drug’s effects (120). Set and setting are thus optimized in the aims of engendering certain types of putatively therapeutic experiences like mystical or “peak” experiences (121).

The third phase of PAP is referred to as integration, a phase that entails patients and therapists meeting after the dosing session (s) to revisit their experiences. The overarching aims of this phase are to process the treatment experiences by, for example, working through distressing content and consolidating insights (90, 116). Much like the preparation phase, actual integration practices are diverse—a recent review identified 10 distinct models of integration which differed in their relative emphasis on mind, body, spirit, lifestyle, relationships, and the natural world (122). The typical duration of this phase is several hours over one to three sessions (90), though PAP practices outside of clinical trials may vary significantly.

In sum, PAP can generally be understood as synergistic mind–body interventions typically conducted in three phases: preparatory sessions, one to two doses of psilocybin administered with careful attention to set and setting, non-directive psychological support, and post-experience integration (80). Actual practices vary significantly, and this is particularly true for psychedelic approaches to ketamine, where the numbers of treatment sessions range from one to dozens (112), dosages range from 0.1 mg up to and beyond 4 mg/kg parenterally (123), and routes of administration include intravenous, sublingual, intramuscular, and oral (112).

#### 2.1.6. Potential advantages for ketamine as a psychedelic

While PAP has demonstrated significant benefits in MDD and TRD (92, 124, 125), there are major knowledge gaps and obstacles to overcome before real-world implementation, which is anticipated to occur within the next several years. For instance, the current psychotherapeutic practices of PAP are heterogenous and relatively ill-defined though there are ongoing efforts to shift to evidence-based approaches like Acceptance and Commitment Therapy (ACT) (118, 126). The external validity of existing PAP trials is also limited by their populations often being highly selected, e.g., by enrolling only the 3–6% of screened patients who are relatively free of suicidality and comorbidity (91, 125). Further, treatment sessions requiring the continual presence of two trained clinicians for upwards of six consecutive hours will inevitably be associated with costs in the range of tens of thousands of dollars per treatment course and other implementation challenges, such as shortages of skilled personnel and infrastructure.

As discussed earlier, ketamine has proven to be somewhat of a chameleon in terms of its utilization in the treatment of depression, with diverse approaches variously resembling ECT, conventional antidepressants, and/or serotonergic psychedelics. In the case of the latter, ketamine has numerous advantages as a putative psychedelic: a long track-record in medicine, excellent evidence as a treatment of severe TRD and suicidality (25, 46), treatment session durations that are approximately 75% shorter (87), minimal drug–drug interactions with a wide variety of medications (psychiatric and otherwise) (127), and safety/efficacy in bipolar affective disorder 1 and potentially depression with psychotic features (41, 128). Psychedelic-like models of care for ketamine thus warrant consideration and study.

In developing the Montreal model, we aimed to create a relatively structured approach to ketamine that capitalizes on its unique strengths, is applicable to complex real-world TRD patients, and is feasible to implement in resource-limited public healthcare systems.

### 2.2. The Montreal model of ketamine for TRD: overview and development

Our efforts toward optimizing ketamine for TRD began with establishing an IV ketamine service employing an ECT-like model of care and relatively standard dosing protocols (39). This approach yielded benefits and limitations similar to those found in the biomedical ketamine literature: rapid improvements in depression and suicidality in approximately 50% of patients, which generally faded within days or weeks of the last dose (25).

In the aim of improving our intervention’s sustained efficacy and tolerability, our multidisciplinary team took advantage of the relative flexibility of a clinical service to implement and evaluate diverse nonpharmacological strategies. We drew significant inspiration from emerging psychedelic research for the actual ketamine dosing sessions, as described in section 2.2.6, but also endeavored to build upon the evidence base for conventional psychiatric and psychotherapeutic interventions where possible.

As summarized in Table 1, our model has come to occupy a middle-ground between biomedical and psychedelic approaches to ketamine. Below, we provide a narrative account of the “lessons learned” from our research endeavors and clinical practice that led to this model’s evolution.

**Table 1:** Comparison of ketamine treatment models: biomedical, psychedelic, and the Montreal model.

Treatment model	Hypothesized mechanism of ketamine’s action	Framing of ketamine’s subjective effects	Protocol components	Treatment characteristics
Biomedical	Neurobiological effects that address neurochemical and structural deficits	Framed as “dissociation”; an adverse effect of treatment “Set and setting” of little importance	Psychiatric and medical assessment Acute treatment course Maintenance	Ketamine administered without accompaniment Subanesthetic dosages (0.5–1.0 mg/kg) Psychoactive effects minimized/mitigated Benefits primarily maintained with additional treatments
Psychedelic	Transformative experiences that, in synergy with neuroplasticity, catalyze growth and recovery	Framed as “psychedelic”; critical for benefits “Set and setting” of essential importance	Assessment Psychedelic preparation Dosing session(s) Integration	Ketamine administered with guides present Doses range significantly Psychoactive effects shaped with various dosages (up to 4.0 mg/kg) and non-pharmacological adjuncts (e.g., blindfolds) Benefits primarily maintained psychologically, e.g., by “integrating” experiences
Montreal model	Neurobiological and psychological benefits that enhance psychiatric care and conventional psychotherapy	Framed as opportunities for experiential learning “Set and setting” of significant importance	Psychiatric and medical assessment Concomitant psychotherapy Rolling psychedelic preparation, treatment, and integration Maintenance	Ketamine administered with clinicians present Subanesthetic dosages (0.5–1.0 mg/kg) Psychoactive effects from relatively low dosing enhanced with non-pharmacological adjuncts (e.g., blindfolds). Benefits primarily maintained with conventional psychiatric care including psychotherapy and occasional maintenance treatments

#### 2.2.1. The need for a “psychotherapy-grade” therapeutic frame

At the clinic’s outset, we proposed trials of three ketamine infusions to appropriate patients with subsequent treatments to be mutually decided upon based on clinical response—much as is done with ECT (20). Unlike our experiences with ECT, however, we found that this lack of a clear therapeutic frame generated numerous issues including anxieties and perverse incentives regarding future treatments. For example, a positive clinical response might be perceived to result in fewer possibilities for treatments and contact with the team, which nearly all patients felt were beneficial. Many early patients thus experienced reemergent symptoms near the end of the treatment course—in other words, “termination reactions,” which serve to illustrate how ketamine differs from psychiatric interventions like ECT.

Termination is generally a subject of some interest in psychiatry but is a critical consideration in psychotherapy (129). For instance, discussing termination is one of the two main activities in the time-limited psychodynamic therapy of James Mann (130), who highlights the importance of exploring the inevitable end of treatment throughout the entire process. This exploration aims to cultivate insight and avoid negative outcomes like regression, ambivalence, and worsening depression, which may be common and significant. The importance of termination and other psychological dynamics in psychedelic therapies are increasingly being recognized (131), mirroring our own experiences that a brief course of ketamine evokes psychological dynamics that more resemble an accelerated short-term psychotherapy than a course of ECT (80).

An essential ingredient for any kind of psychotherapy is establishing a clear and consistent therapeutic frame. This includes: a shared understanding for the treatment activities, the schedule of sessions, and clear parameters for communication and follow-up care (132). This structure is not just necessary for psychotherapeutic activities, it is also understood to be therapeutic in its own right (130, 132).

While most clinical research entails a solid therapeutic frame by virtue of the research protocol, explicit efforts are required in clinical settings. Our experiences suggest that establishing a “psychotherapy-grade” therapeutic frame is vital for safety and efficacy with ketamine, particularly given the complexity and comorbidity of TRD with conditions like personality disorders (4). Not only can such a frame reduce uncertainty and mitigate termination reactions, it provides the structure needed for psychological exploration.

#### 2.2.2. The hype bubble: managing expectations

Patients referred to our service often (and increasingly) arrive with unrealistically high expectations for ketamine, anticipating powerful or even miraculous benefits (133). This clinical phenomenon, paralleling the broader psychedelic “hype bubble” (86), poses challenges and risks.

Many of our early patients—even those who reported significant benefits with ketamine—were disappointed with a lack of life-changing effects and/or by their transience. This disappointment can readily lead to discouragement, hopelessness, and even increased suicidality, particularly in patients who have already experienced years of past treatment failures.

To mitigate these risks, we began systematically incorporating explicit discussions about treatment expectations during all initial evaluations, aiming to foster more moderate outlooks and greater feelings of agency from the very outset. This includes, for instance, emphasizing the importance of behavioral changes (discussed below), establishing “backup plans” to be undertaken in case ketamine should prove unsuccessful, and generally shifting perceptions of ketamine from being a panacea that is passively received toward being one important component of a multifaceted and active effort toward recovery.

Such conversations can significantly mitigate eventual disappointment and hopelessness—especially when undertaken before the ketamine treatments have begun.

#### 2.2.3. Behavioral windows of opportunity: establishing SMART treatment goals and valued directions

As we began establishing clear therapeutic frames and exploring treatment expectations on evaluation, we evolved toward explicitly presenting ketamine as follows: an intervention that can yield benefits that may well be significant, yet are likely to be transient. Given our lack of resources for maintaining benefits by frequently repeating doses, this framing naturally led to direct conversations with patients about how a resultant “window of opportunity” of symptomatic improvement [and potentially enhanced neuroplasticity (96)] might be best utilized. In other words, what activities might be undertaken during—but also before, and after—the ketamine treatments to deliver maximal long-term benefits.

Similar questions pertain to the preparation and integration phases of psychedelic therapies. However, as experts have commented, the psychedelic literature on preparation and integration can be relatively vague and inconsistent regarding what these practices should entail (122, 126). We thus instead drew from wider bodies of knowledge about the relationship between behaviors and psychological wellbeing, as well as the well-established practices of goal setting in conventional psychotherapies like CBT and ACT.

CBT, the psychotherapy with the greatest evidence-base in depression (17), has been proposed by experts as the most obvious “default” approach for psychedelic therapies (134). One of CBT’s essential components is the collaborative development of personalized (and often behavioral) goals (135). The therapeutic value of such behavioral goals is further supported by evidence for Behavioral Activation Therapy (BAT) (136). BAT is a relatively simple, short-term therapy that partially overlaps with CBT but focuses almost entirely on pursuing adaptive behavioral changes. According to a 2020 Cochrane review of 53 studies with 5,495 participants (136), BAT is at least as effective in depression as CBT or any other approach that it has been compared to.

Based on these therapies and their extensive evidence, our model incorporates behavioral goal-setting into evaluation and preparation using the common “Specific Measurable Achievable Relevant Timely” (SMART) framework (135). Patients and our clinicians establish at least three mutually acceptable SMART goals to be initiated before beginning ketamine treatments and worked on throughout. These generally focus on evidence-based lifestyle changes, such as: improving sleep hygiene (137), improving diet (138), establishing consistent routines (139), engaging in meaningful social activities (140), and/or increasing levels of physical activity (141). Common goals include engaging in 20 min of daily walking and establishing fixed sleeping hours, which are modest yet highly relevant to our populations of patients with TRD who frequently report highly irregular routines and extreme behavioral deactivation.

For patients with healthier lifestyles and habits, either on evaluation or as the treatment progresses, our goal-setting process evolves to more resemble the “committed action” component of ACT. I.e., treatment goals become less about engaging in evidence-based lifestyle changes, and more about pursuing meaningful actions that align with an individual’s unique values (126). These ACT-style goals are often more abstract than improving sleep hygiene or engaging in exercise, for instance, yet generally remain well-suited to the specificity and clarity that the SMART framework provides.

Our experiences suggest that the ketamine “treatment situation” is an ideal opportunity for such behavioral modifications. Patients’ high expectations for ketamine and desires to optimize their chances for response can translate into high levels of engagement in pursuing changes—which is also best initiated early in the ketamine treatment process. In turn, this collaborative goal-setting process can help patients cultivate a healthier locus of control, as well as insight into the psychological obstacles that contribute to their struggles.

#### 2.2.4. Pharmacologic windows of opportunity: optimizing psychiatric medications

Extending our observations that most patients readily engaged with difficult but potentially beneficial behavioral goals, we hypothesized that the ketamine treatment process might also be an opportunity for difficult but potentially beneficial medication changes. Of many such potential changes, our most common recommendation has been to reduce the use of benzodiazepines and related sedatives (BZDRs).

For depression, guidelines only recommend short-term benzodiazepines given evidence for tolerance (142), potential harms (143), and questionable long-term efficacy (144). Nevertheless, approximately half of the patients referred to our service actively receive long-term BZDRs—often at high doses (145). In many cases, patients report no clear indication for these treatments, nor being adequately informed about their potential harms. Given their risks and especially the emerging (if conflicting (146)) evidence that benzodiazepines may dampen or shorten the antidepressant effects of ketamine (147), we consistently propose a trial of BZDR discontinuation to all patients. This typically entails a slow taper such that final doses occur within several days of the first ketamine treatment.

As with the pursuit of lifestyle goals, patients tend to readily engage in such processes when provided with education and support. Our recent results from a cohort study of TRD patients receiving ketamine in our model found that all were willing to attempt BZDR discontinuation, almost all achieved total abstinence during treatment, and the majority remained BZDR-free after an average follow-up of 1 year (145). These results are particularly noteworthy given our population’s severity, and that only one prior study that has even attempted BZDR discontinuation in patients actively suffering from depression (148). In addition to preclinical and clinical evidence suggesting that ketamine can mitigate psychological BZDR withdrawal symptoms (149–151), we have found that the initiation of a course of ketamine is an excellent psychological opportunity to undertake rational medication changes (145).

#### 2.2.5. Concomitant psychotherapy: psychological accompaniment during a critical period

In addition to optimizing the lifestyles and pharmacological treatment of our patients, we began routinely requiring that our patients engage in at least one-hour of weekly evidence-based psychotherapy beginning at least 2 weeks before their ketamine treatments. The impetus for this addition was multifold:

1. Significant evidence suggests that psychotherapy has additive benefits with pharmacological treatments like oral antidepressants—and indeed can sustain the benefits of antidepressants after they are discontinued (80, 152). Although more research is needed, it is reasonable to suppose the same would apply to ketamine, especially given its putative neuroplastic effects (95).
2. Ketamine treatment experiences can generate emotionally-charged and potentially distressing psychological content, in addition to disappointment and interpersonal dynamics as discussed above. Without adequate follow-up, such content can be harmful or treatment-interfering; with it, this content can give rise to fruitful psychotherapeutic exploration.
3. Ketamine’s unique and rapid benefits, especially against suicidality (49) and the cognitive impairments of depression (48), should theoretically reduce obstacles to productive psychotherapy. Indeed, anecdotally, many of our patients’ therapists have reported increased engagement with their patients during and after their course of ketamine treatments.
4. Patients who are benefitting from this concomitant psychotherapy may be able to continue after the ketamine treatment process, as resources and needs dictate, providing a comparably inexpensive and safe form of maintenance therapy.

Psychedelic trials similarly incorporate therapy sessions outside of drug treatment sessions, but these are almost always conducted by the same clinicians (116). Partly due to resource constraints, we are intentionally flexible regarding these therapists, who may be external therapists from our hospitals, referring psychiatric services, or the community, or learners rotating in the service. Some such therapies are in place prior to evaluations, and others are arranged to begin alongside the ketamine process. As such, our model does not specify the exact type of therapy, but rather requires any evidence-based approach, such as CBT or ACT, that a particular patient is willing to pursue.

This use of concomitant psychotherapy occurring alongside—but somewhat independently of—ketamine treatments is atypical for psychedelic approaches but very common in existing psychiatric models of care. I.e., patients being treated by psychotherapy plus conventional medications and/or neurostimulation typically receive care from multiple clinicians, who (ideally) work in close collaboration.

The flexibility afforded by this approach has obvious feasibility benefits, such as alleviating the need for all treating clinicians to be experts in both conventional psychotherapy and ketamine/psychedelic approaches. It also aligns well with extensive evidence that therapist-patient alliances are more strongly related to outcomes than are technique specifics in depression (153). In other words, one may generally expect more benefits from prioritizing concomitant psychotherapy characterized by strong therapeutic alliances than by specific techniques.

In cases where a patient’s comorbid conditions suggest that a particular technique is particularly likely to be beneficial—e.g., trauma-focused therapy for comorbid PTSD (154)—the model’s flexibility also allows for prioritizing a certain therapeutic approach. This is an important benefit given that TRD populations have high rates of diverse comorbidities (7).

In our experiences, the various benefits of implementing psychotherapy in a concomitant fashion generally outweigh the potential drawbacks, provided that ketamine clinicians and psychotherapists work in close coordination.

#### 2.2.6. Psychedelic-inspired treatment sessions

The aforementioned aspects of the Montreal model largely pertain to what happens outside of actual ketamine treatment sessions. They were mostly inspired by conventional psychiatric evidence and may thus hold relevance for even purely biomedical approaches to IV ketamine. In contrast, the following sections describe our approach to the actual ketamine dosing sessions, which has largely been informed by the psychedelic paradigm.

As discussed, there are conflicting biomedical and psychedelic views about the altered states of consciousness engendered by ketamine: are they inconvenient treatment side-effects, or are they essential drivers of psychological benefits? Having administered hundreds of treatments with diverse contexts and framings, our view lies between these extremes. We have come to see ketamine experiences as neither unimportant nor as essential, but rather as valuable opportunities for experiential learning when paired with appropriate settings and mindsets.

##### 2.2.6.1. Reconciling the biomedical and psychedelic ketamine paradigms: the analogy of dreams

Since its discovery, ketamine’s unique psychoactive effects have inspired countless descriptors. Indeed, before the label “dissociative” was chosen, the term “dreaming” had been proposed by its creators (155).

In the decades since, the anesthesia literature has found utility in both terms: while ketamine is typically described as a “dissociative anesthetic,” the phenomena of “ketamine dreams” during anesthesia have also received significant attention (156–158). In psychiatric contexts, dreams can similarly serve as powerful analogies for understanding ketamine’s multifaceted effects and reconciling its divergent bodies of literature.

Much as dreams often contain bizarre transformations of memories and emotions, ketamine experiences are often described as visions of current and past events juxtaposed with perceptual distortions (87, 112, 159). Experiences of death and dying, well-known to occur in dreams, are also more common with ketamine than any other psychotropic drugs (160, 161). And, just as the meaning and interpretations of dreams varies dramatically across cultures and contexts—and indeed, amongst schools of psychotherapy (162, 163)—the personal significance of ketamine experiences is shaped by their framings.

When introduced as pathological or meaningless, patients are prone to dismissing ketamine experiences akin to hastily forgotten dreams (158). Conversely, just as patients report enhanced dream recall and significance when working with dream-oriented psychotherapists (163), framing ketamine experiences as potentially meaningful can increase their attributed importance and clarity. This may arise from a clinician’s explicit statements or by their implicit communications, including simple demonstrations of curiosity toward a patient’s ketamine experience.

In our services, we gradually evolved away from presenting ketamine’s effects as relatively meaningless dissociation-like experiences toward being more reflections of one’s mind and one’s contexts. This shift yielded a double-edged sword. On one hand, such framings can result in reports of more vivid ketamine experiences characterized by greater personal significance, perceived insights, and emotionality. On the other hand, distressing experiences such as near-death experiences can be all the more distressing when they have been framed as meaningful rather than somewhat random or insignificant.

Although further study is needed, we have found that presenting ketamine experiences as being reflective of one’s inner landscape is consistent with their phenomenology and is beneficial overall. Experiences characterized by autobiographical memories, bizarre symbolism, and even recent media exposure can all be sources of catharsis and/or insight (164)—but only with supportive contexts and adequate psychological support.

##### 2.2.6.2. The importance of treatment settings

We have administered ketamine in settings ranging from dedicated outpatient spaces (resembling the living-room like environments of psilocybin trials) (90), to barren inpatient facilities. As has been described with serotonergic psychedelics and esketamine (90, 165), our experiences have repeatedly confirmed the value of calm, de-medicalized rooms toward reducing the distress associated with altered states of consciousness.

Perhaps more importantly, the simple use of blindfolds can dramatically alter patient experiences. By reducing the external stimuli that ground patients in their physical environments, patients describe blindfolds as greatly increasing the degree and intensity of perceptual changes induced by ketamine. Likely as a result of blindfolds and other contextual factors, and in contrast to others (166), we have consistently found that subanesthetic dosages of ketamine, e.g., 0.5 mg/kg, can produce powerfully psychedelic experiences including high scores on the Mystical Experience Questionnaire (164, 167).

If blindfolds increase the subjective intensity of ketamine experiences, music can significantly influence their content (168). As we have reported (169), patients who have received ketamine infusions with and without music describe that music can generate vivid synesthetic visions, reduce anxiety, and support exploration of ketamine experiences. These powerful impacts motivated our recently completed RCT where 32 patients were randomized to receive music or matched non-music support (e.g., engaging in relaxation or simple mindfulness exercises) during their course of ketamine treatments (13).

The results of this trial, currently under peer-review, demonstrate that music can dramatically alter patient experiences. Indeed, based on our patients’ reports, we have come to see the selection of music as of equal importance to the selection of a psychotherapeutic approach, in line with music being analogized as a “hidden therapist” in psychedelic studies (94). Our process for doing so is described below.

###### 2.2.6.3. Experiential learning: process over content

Administering ketamine in psychedelic-like contexts—e.g., with blindfolds, music, and some expectation for meaningfulness—has important clinical and ethical considerations. For instance, false memories of past traumas have been well-described in relation to various mind-altering substances (170), and patients may experience more disappointment when expecting profound mystical experiences versus transient dissociation-like experiences (86).

Partly to mitigate potential harms of false memories or other unintended consequences of heightened suggestibility, partly to reduce distress associated with challenging or disappointing experiences, and partly because of greater perceived relevance for the day-to-day lives of our patients, we have come to emphasize *how* a particular patient experiences a ketamine treatment more than *what* it may have evoked. In other words, we have found that a treatment’s process makes for a superior therapeutic focus versus its content.

This view is well-aligned with CBT and ACT models of psychedelic experiences as powerful opportunities for experiential learning, such as “learning to let go” (126, 171). I.e., that the practice of navigating all varieties of psychedelic experiences—bizarre, autobiographical, joyful, terrifying, and so on—can translate into increased capacities for acceptance and reduced needs for experiential avoidance beyond the treatments. These active practices have clear significance for day-to-day living with depression and are arguably better supported by non-psychedelic psychotherapy research (126, 171, 172) than, for instance, more passively receiving a mystical experience.

In addition to validating evoked emotions, our clinicians thus orient all phases of the ketamine treatment process toward cultivating curiosity for one’s current-moment experience, “defusing” challenging thoughts (171), and taking value-driven actions. For instance, each treatment is begun with brief exercises like body scans or the mindful focusing of attention on the breath. Patients are encouraged by their clinicians to avoid suppressing emotions or labeling sensations with judgmental terms, but rather to fully experience them without becoming entangled in their associated cognitions. The same orientation is encouraged throughout the actual ketamine experiences, and similar exercises are recommended between sessions.

In essence, our psychological approach can be summarized as gentle encouragement to: *feel emotions, defuse thoughts, and change behaviors*. In alignment with the establishment of behavioral goals at the treatment’s outset, the aim is to facilitate experiential learning with ketamine treatments toward cultivating engaged, collaborative, and flexible life orientations as a corrective force to the passivity, rigidity, and nihilism, commonly found in TRD (78).

###### 2.2.6.4. Psychedelic experiences as amplifiers of conventional psychotherapeutic mechanisms

A common (if unsubstantiated) description of psychedelic experiences is that one or few experiences can provide the equivalent psychological benefits of years of talk therapy (173). How this might occur, however, remains uncertain.

In this subsection, we share preliminary hypotheses based on clinical observations, quantitative and qualitative research of our patients regarding the psychological mechanisms that may underly psychedelic ketamine experiences. These are presented in Table 2 and related to known mechanisms of conventional psychotherapy where applicable. Although further research is required, we suggest that these processes are not specific to ketamine, nor to TRD, but may rather be expected to arise in any therapeutic clinical situation where a patient receives a powerful psychoactive drug with psychological accompaniment.

**Table 2:** Postulated psychological therapeutic mechanisms underlying ketamine and other psychedelic drug experiences.

Mechanisms found in conventional psychotherapy	Exposure	Exposure to thoughts, sensations, and emotions that may be otherwise avoided has been proposed as a “transdiagnostic component of [all] successful psychotherapies” (174). This process may be explicit, as in behavioral treatments of phobias (154), or more implicit, as in explorations of conflictual feelings in psychodynamic psychotherapy (175). Psychedelic experiences can offer powerful opportunities for such exposure in that they almost universally provide vivid experiences of challenging internal content (134).
	Experiential learning	Successfully engaging in a therapeutic process can impart skills and learning that extend beyond the specific content that may have been addressed. For instance, a fundamental goal of CBT is for patients to learn to “become their own therapists,” applying various skills to diverse situations (176). Similarly, navigating the distressing or bizarre content of psychedelic experiences can impart (or even condition) skills for increasing “acceptance” of distressing internal content (171). This particular mechanism against experiential avoidance is attracting significant attention, and indeed has been recently operationalized in the Acceptance/Avoidance-Promoting Experiences Questionnaire (177).
	Reparative interpersonal interactions	A core common factor of psychotherapy is a strong patient-therapist relationship (153), which is essential for collaboration and a key source of corrective interpersonal experiences. Psychedelic treatments entail even more vulnerability and thus require even more trust than conventional psychotherapy. This may serve to magnify the impact of reparative interpersonal interactions, as well as potential interpersonal harms (131).
	Increasing understanding of, and interest in, one’s own mind	Many psychotherapies aim to increase awareness of previously underappreciated aspects of one’s mind, e.g., by exploring “core beliefs” in CBT and the meanings of dreams in psychodynamic therapy (163, 176). Psychedelic experiences, practically by definition, can involve vividly experiencing aspects of one’s mind that are otherwise beyond awareness. Such experiences may bestow particular insights (178), and may also generally enhance curiosity toward one’s inner world.
	Decreasing excessive or maladaptive certainty	Conventional psychotherapies aim to revise maladaptive beliefs in various ways, including engaging in behavioral experiments (136), directly challenging cognitions (176), and cultivating distance from thoughts (126). Psychedelic experiences may similarly decrease certainty in prior beliefs, simply by virtue of having lived a radically different form of consciousness or emotional state, or by direct pharmacological actions as postulated in the Relaxed Beliefs Under Psychedelics (REBUS) model (179).
	Emotional release	Although their conceptualizations vary widely, nearly all psychotherapies entail the mobilization and expression of emotions toward therapeutic ends (180). Psychedelic drugs are well known to evoke strong emotions, and certain emotional experiences—emotional “breakthroughs”—are amongst the most robust predictors of psychedelic benefits (104).
Relatively unique psychedelic mechanisms	Temporary reprieve from suffering	Patients living with chronic psychiatric disorders often experience prolonged and uninterrupted periods of suffering. Temporarily experiencing a profoundly *different* state, even if strange or meaningless, can offer a beneficial “pause”—albeit one that may be linked to the development of addiction or pro-avoidance beliefs, as has been observed with psychedelics (177).
	Connectedness, awe, and mysticism	A growing body of literature has reported on relatively unique features of psychedelic drug experiences, including heighted feelings of connectedness (181), of awe (182), and (potentially relatedly) mystical experiences (167), all of which have been linked to clinical benefits.

###### 2.2.6.5. Psychedelics as amplifiers of patient preferences

As mentioned above, the Montreal model was recently evaluated in an RCT examining the effects of music on ketamine infusions in patients with TRD (13). Two key themes to emerge repeatedly from conducting this study were the importance of patient preferences and the therapeutic value of choice.

A key difference between our protocol and those employed in most PAP studies is our use of six relatively brief ketamine infusions over 4 weeks, rather than one or two longer psilocybin sessions (91, 92). This dosing protocol—largely inspired from the biomedical ketamine literature—has turned out to provide unique opportunities for iterative treatment tailoring. For instance, participants can be actively engaged in selecting each treatment’s music based on their previous treatment experiences and their current-moment preferences, rather than relying on a standard playlist for all patients as in most psychedelic studies (90, 94).

Meta-analytic evidence demonstrates that incorporating patient preferences into clinical processes can improve outcomes (183, 184). This may be especially true for psychedelic treatments, given that these drugs can powerfully enhance the influence of contexts. For instance, an individual’s slight typical preference for a particular playlist over another can be magnified by psychedelic substances into radically different experiences. From this angle, there can be no “best” musical playlist for psychedelic therapy, but rather a best choice for a given patient on a given day, and the same may be said of many elements of a psychedelic treatment.

We thus take advantage of our six treatment sessions to incorporate patient choice at every opportunity, within the boundaries of the therapeutic frame. Beyond the co-selection of music, this includes collaborative selection of behavioral goals and the nature of psychological support provided during ketamine treatments, which may include quiet accompaniment, breathing exercises, or guided body scans. Many of our patients have reported that such adaptations have both direct benefits—e.g., finding music that’s well-suited to a given treatment—as well as indirect benefits, such as increasing their feelings of engagement and autonomy.

###### 2.2.6.6. Building momentum: rolling preparation, treatment, and integration

Our model’s six intravenous ketamine treatments provide patients with a similar amount of time experiencing an altered state of consciousness as two doses of psilocybin (91, 124, 125). However, this time is spread out over 4 weeks rather than one or two. This pattern of shorter but more frequent doses over longer time periods gives rise to distinct temporal dynamics.

Rather than the three distinct PAP phases of preparation, treatment, and integration, we describe our model’s equivalent treatment phases as “rolling”: each treatment serves as preparation for the subsequent treatment and integration of the previous. An experience characterized by difficulties in “letting go,” for instance, can be revisited in subsequent treatments toward gradually building capacities for acceptance (169). Similarly, feelings of disappointment with an experience’s content or its after-effects can be translated into more realistic expectations for subsequent treatments. In turn, patients often report revisiting disappointment or distressing content from previous sessions with greater equanimity, clarity, or emotional catharsis.

In this way, our therapeutic process more resemble the gradual exploration and skill-building of conventional psychotherapies such as CBT (171), rather than breakthrough or mystical models of PAP (104, 105). Although more research is needed, we believe that this slower-but-steadier endeavor is better suited to complex patients suffering from severe and chronic TRD given that six treatment visits provide multiple opportunities to become engaged in the treatment process, revisit behavioral goals, and consolidate insights.

## 3. Results: the Montreal model of ketamine for TRD

**Figure 1:**
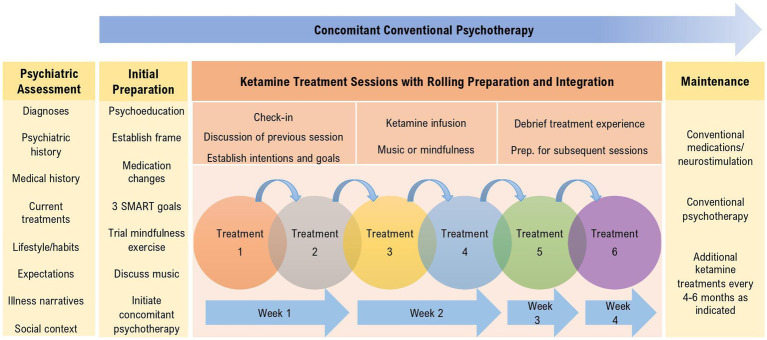
Overview of the treatment components and session activities of the Montreal model of ketamine for treatment-resistant depression.

Having reviewed the underlying rationale, in the following sections we present the four phases of the treatment model, summarized in Figure 1.

### 3.1. Psychiatric referral and assessment

Given the significant rates of psychiatric and medical comorbidity in TRD (7), a thorough 90-min psychiatric assessment is an essential first-step in our model [in addition to standard medical investigations (39)]. Its key aspects, summarized in Table 3, include confirming psychiatric diagnoses, reviewing current and past treatments, and eliciting current-moment lifestyle factors.

**Table 3:** Psychiatric assessment aspects and rationales.

Aspect	Key variables	Rationale
Psychiatric diagnoses	Unipolar or bipolar depressive disorder Comorbidity including personality traits and disorders Active suicidality	To confirm indication and inclusion/exclusion criteria (see Supplementary Information). Of note, our major exclusion criterion is substance use, given the potential for iatrogenic harms. We do not exclude patients with comorbidities such as personality disorders, insofar as these do not pose major safety concerns or interfere with forming adequate working alliances. Similarly, acute suicidality is not an exclusion criterion, but does necessitate active exploration and safety planning.
Past psychiatric treatment history	Past medication, neurostimulation, and psychotherapy trials including duration, adherence, and response	To confirm and stage resistance [e.g., using the Dutch Method (185)], elicit potentially beneficial medication changes, and establish “backup” treatment plans should ketamine prove to be ineffective.
Current psychiatric treatments	Medications, psychotherapy, and neurostimulation	To identify potentially beneficial medication changes and ensure concomitant psychotherapy for the ketamine treatment process
Medical history and treatments	Neural, cardiovascular, renal, and hepatic health Conditions commonly associated with depression such as thyroid disorders, anemia, and obstructive sleep apnea	To ensure physical safety with ketamine and to address medical conditions that may be contributing to depressive symptoms, particularly obstructive sleep apnea (137).
Lifestyle and habits	Substance use Sleep Exercise Routine Diet Daily activities	To evaluate risk for substance abuse and identify key areas for evidence-based psychoeducation and treatment goals.
Current psychosocial situation	Current stressors Important social supports	To ensure adequate support for an intensive treatment process and to orient psychosocial interventions.
Illness narratives	Patients’ understanding of the causes of their depression and their potential resolutions	To better understand an individual’s sense of their own illness such that empowered, collaborative therapeutic narratives may be co-created.
Expectations	Patients’ understanding of how ketamine may be therapeutic and their potential role in the treatment	To elicit and potentially mitigate unrealistic or overly passive expectations for treatment.

### 3.2. Initial preparation and concomitant psychotherapy

For a patient deemed to be a good candidate, two to three preparatory sessions ranging from 30 to 60 min in length are undertaken prior to beginning ketamine treatments, as summarized in Table 4. These sessions are guided by the information gathered during evaluation and aim to establish a shared understanding of ketamine’s effects and the treatment process as detailed in section 2.2. I.e., that ketamine can potentially provide “windows of opportunity” of symptomatic relief from depression and psychologically beneficial treatment experiences, both of which can facilitate longer-term efforts toward recovery.

**Table 4:** Goals of preparation, in approximate order.

Goal	Details
Establishing a treatment frame	Agreeing upon treatment details including the number and timing of sessions, as well the parameters for communication and follow-up.
Cultivating a strong therapeutic alliance	Exploring recent and past life events with empathy and curiosity.
Psychoeducation and management of expectations	Presenting ketamine as an off-label treatment, potentially acting through enhanced neuroplasticity, which may yield significant if transient psychiatric benefits.
Determining behavioral treatment goals	Collaboratively determining at least three evidence-based, SMART behavioral goals to be pursued before, during, and after the ketamine treatments such as improving sleep hygiene practices, engaging in regular exercise, and pursuing valued action.
Optimizing medications	Deciding upon and initiating potentially beneficial medication changes such as a trial discontinuation of benzodiazepines or switching ineffective antidepressants.
Framing the ketamine experience	Presenting ketamine experiences as person-specific and context-dependent, which range greatly in their content and are variously described as dissociative, mystical, and/or dream-like. Emphasizing the importance of process over content—e.g., that the treatment experiences provide opportunities for cultivating curiosity and acceptance.
Trial mindfulness exercise	Undertaking a brief mindfulness exercise such as body scanning or thought defusing in alignment with the aim of experiential learning during ketamine experiences.
Music discussion	Exploring an individual patient’s relationship to music and their preferences, to determine which music (if any) will be used for the first treatment.
Arranging for concomitant psychotherapy	Discussing and arranging for at least 1 h weekly of evidence-based psychotherapy to be initiated prior to the ketamine treatments beginning.

### 3.3. Psychedelic-like ketamine treatment sessions: rolling preparation, treatment, and integration

The actual ketamine treatment course typically entails six infusions over the course of 4 weeks at standard dosages: 0.5 mg/kg infused over 40 min, twice per week for 2 weeks then once weekly for 2 weeks. These roughly 2-h sessions occur in comfortable and (ideally) esthetically pleasing dedicated rooms, with at least one clinician present throughout to ensure adequate safety monitoring and accompaniment.

These treatments are administered with hallmarks of the psychedelic paradigm: blindfolds, “rolling” integrative and preparatory psychological support, and music and/or mindfulness exercises. Ketamine experiences are framed as being meaningful in terms of their process, and potentially their content. Although the stated emphasis of these sessions is on experiential learning, clinicians are mindful of other known and hypothesized psychotherapeutic mechanisms summarized in Table 2, such as emotional release. Sessions follow a structured course as per Table 5.

**Table 5:** Ketamine treatment session activities, in order.

Activity	Description
Check-in	Any recent life events, medical/psychiatric risk, symptomatic changes, changes of treatments, experiences in concomitant psychotherapy, and progress on treatment goals since the previous visit are reviewed. The treatment’s eventual termination is discussed explicitly, particularly during the last treatments of the course.
Rolling integration	The experiences of the last ketamine treatment (if relevant) are discussed, with an emphasis on particularly meaningful content, the activities used to “integrate” the experience, and their relevance of the experiences toward day-to-day life.
Establish intentions and activities for the current session	Goals for the current ketamine treatment are mutually agreed upon, including what, if any, music will be employed (including listening to brief excerpts where desired) and the degree of guiding from the clinicians.
Ketamine initiation	After ensuring satisfactory baseline hemodynamic parameters, psychological readiness, and consent to proceed, an intravenous line is installed and the infusion is started.
Ketamine infusion	A brief mindfulness exercise is undertaken, consisting of observing and cultivating curiosity for one’s mind, one’s breathe, and any notable physical sensations. For sessions conducted with music, the selected playlist —typically 50–60 min—is then begun via both headphones and speakers, which usually limits further interaction until its completion. For sessions conducted without music, clinicians gently encourage patients to practice openness and acceptance to whatever the experience evokes, following the patient’s lead and checking-in at least every few minutes. Blood pressure is measured at 10–15 min intervals and both heartrate and oxygen saturation are monitored throughout.
Debrief	Following the completion of the 40-min infusion or the music playlist (if music has been employed), clinicians engage in discussion with patients about their experiences for about 20–30 min. The therapeutic stance prioritizes sitting with and validating any strong emotions, highlighting moments of success or difficulties in “letting go,” and revisiting prior intentions and past ketamine experiences.
Rolling preparation	Plans for the next days and the subsequent treatment are reviewed and discussed, including the established behavioral goals.
Discharge	Once patients are psychologically and physiologically ready, with vital signs within normal limits, they are discharged with reminders of safety considerations and encouragement to contact the ketamine clinicians in case of adverse reactions or significant distress.

### 3.4. Follow-up and maintenance

A major impetus for the development of the psychological aspects of the Montreal model has been the goal of minimizing the need for ongoing ketamine treatments, which can be burdensome, costly, and potentially associated with long-term psychological and physiological harms (32, 33, 78). Although more research is needed, our experiences and emerging results suggest strong maintenance of benefits in many cases. For instance, we have recently reported the case of a patient who has remained remitted from severely refractory TRD (and withdrawn her requests for medical aid in dying for depression) since several years (12).

Maintenance of benefits in our model takes three forms, tailored to patients according to their unique trajectory, comorbidity, and psychosocial circumstances as detailed in Table 6: medication/neurostimulation, concomittant psychotherapy, and maintenance ketamine treatments. In alignment with our conception of ketamine as not being a standalone treatment, but rather a catalyst for multifaceted psychological and psychiatric care, additional ketamine sessions are only one component of maintenance therapy in the Montreal model. As presented to patients on first evaluation and in alignment with all treatment phases, the maintenance phase emphasizes the importance of behavioral changes and continual collaborative efforts in the pursuit of lasting recovery from TRD.

**Table 6:** Forms of maintenance therapy and their rationale.

Form	Rationale
Medication/neurostimulation	The vast majority of our patients receive conventional medications before, during, and after the ketamine treatment process. Although a patient’s regimen might be optimized in advance of the ketamine treatments—e.g., by a trial discontinuation of benzodiazepines—we generally anticipate that the severity and chronicity of our TRD population will necessitate long-term medications like antidepressants and mood stabilizers, most of which all highly compatible with ketamine. Additionally, some patients opt to initiate or continue neurostimulation treatments such as transcranial magnetic stimulation.
Concomitant psychotherapy	A major benefit of the Montreal model’s concomitant psychotherapy is the flexibility that is afforded by having patients followed by psychotherapists semi-independently of the ketamine treatment process. As described above, where indicated and available, this allows patients to continue their conventional concomitant psychotherapy at regular or as-needed frequencies.
Maintenance ketamine treatments	We offer one-off ketamine maintenance sessions for responders at a frequency of approximately every 4–6 months. The rhythm of these sessions resembles the practice of psychotherapy “booster” sessions, which have been shown on meta-analysis to successfully maintain psychological benefits of therapy at a frequency of roughly every 3–6 months (186). These sessions are conducted much like acute treatments, including an emphasis on revisiting behavioral goals and experiential learning.

## 4. Discussion

Depression, particularly when refractory to current treatments, represents a significant global health challenge. The (re-)emergence of ketamine and serotonergic psychedelics as potential treatments for depression is generating considerable interest. However, uncertainties remain regarding the integration of these innovative treatments into existing psychiatric services—standardized, resource-efficient treatment models represent a pressing need.

In this article, we present the Montreal model of ketamine for TRD, a biopsychosocial approach developed gradually over years of real-world clinical experiences and research endeavors. Our aim has been to help bridge the current divide between biomedical and psychedelic models of ketamine while aiding in the interpretation of our recent and upcoming research results (12, 13, 145, 164, 169). We have thus endeavored to provide sufficient information for researchers and clinicians from diverse backgrounds to understand our approach, from its theoretical underpinnings up to its session activities and clinical implementation.

For researchers and clinicians most familiar with biomedical perspectives on ketamine, we suggest that the incorporation of psychological treatment adjuncts may feasibly address ketamine’s major limitations. This includes establishing clear therapeutic frames, leveraging the increased motivation and the windows of opportunity that the ketamine treatment situation can provide, and delivering ketamine with contexts that permit psychedelic-like experiential benefits. As we have described throughout the article, these psychological enhancements may improve the tolerability and sustained efficacy of ketamine, offering a resource-efficient alternative to frequently re-administering doses.

For those familiar with the psychedelic paradigm, we have reviewed aspects of conventional psychotherapy research and presented our experiences conducting a form of psychedelic therapy in real-world psychiatric contexts. The challenges and potential solutions described above may hold relevance to for the upcoming transition from clinical trials to clinical care that drugs like psilocybin face. We also highlight potential advantages that psychedelic-ketamine models offer over serotonergic psychedelics such as psilocybin. Our model’s six ketamine treatments yield approximately the same amount of time in an altered state of consciousness as 12 psilocybin treatments but spread out over multiple sessions. The associated “rolling” therapeutic rhythm may better suited to managing expectations, navigating psychological challenges, and supporting behavioral changes in highly refractory cases. Other relatively short psychedelic-like drugs, like N,N-Dimethyltryptamine (DMT) and nitrous oxide, may be readily employed in similar models.

Yet the uniqueness of ketamine extends beyond its short duration of action—it is also arguably the most forgiving putative psychedelic. As the biomedical ketamine literature demonstrates, its psychoactive effects can be distressing when administered in medical environments (79), but at nowhere near the degree that would be expected from psilocybin or other classical psychedelics in such contexts—as demonstrated by the near ubiquitous distress seen in the early psilocybin and LSD psychotomimetic literature (100). Ketamine is also arguably much better suited for suboptimal real-world contexts given its meta-analytic evidence for safety and benefits even without psychological support, and in highly severe patients—including those with active suicidality and bipolar disorder (41, 46). Indeed, our recent RCT (manuscripts currently under review) investigating music with ketamine found both the music and non-music interventions to be effective and tolerable even in a population characterized by high rates of comorbidity, suicidality, and chronicity (12). Based on preliminary scientific evidence in the literature, we believe that the Montreal model of ketamine may prove to be valuable in other populations, such as patients grappling with depression in the final stages of life (187).

In conclusion, we join with experts calling for future research into the psychological aspects of depression and TRD (78), beyond ketamine and psychedelics. Just as criticisms of “biological reductionism” and “psychedelic exceptionalism” have been raised regarding the respective biomedical and psychedelic literatures (188, 189), the broader field of depression research would benefit from more multifaceted and integrative approaches. This article, in alignment with the Montreal model, endeavors to consider how overlooked facets of depression, such as maladaptive behavioral patterns and psychological dynamics, interact with pharmacology. Future research should explore and dissect the psychological factors incorporated in the Montreal model in the pursuit of treatment models that empower patients to create change and cultivate a sense of agency in their recovery.

## Data availability statement

The original contributions presented in the study are included in the article/supplementary material, further inquiries can be directed to the corresponding author.

## Author contributions

NGa: Conceptualization, Methodology, Supervision, Writing – original draft, Writing – review & editing, Funding acquisition. JD: Conceptualization, Methodology, Supervision, Writing – original draft, Writing – review & editing. JT-L: Writing – review & editing, Conceptualization, Data curation. NGo: Writing – review & editing, Conceptualization. AL: Writing – review & editing. KL: Writing – review & editing. DE: Writing – review & editing. SD: Writing – review & editing. GT: Supervision, Writing – review & editing. SR: Conceptualization, Supervision, Writing – original draft, Writing – review & editing, Funding acquisition, Methodology. SR-D: Supervision, Writing – original draft, Writing – review & editing, Funding acquisition. KG: Conceptualization, Supervision, Writing – original draft, Writing – review & editing, Data curation, Funding acquisition, Methodology.

## Funding

The author(s) declare financial support was received for the research, authorship, and/or publication of this article. This work was funded by the Reseau québécois sur le suicide, les troubles de l’humeur et les troubles associes (RQSHA), as well as unrestricted charitable funds from the Doggone Foundation.

## Conflict of interest

AL has received grant support from MED-EL and Oticon Medical. DE has received funding from NIHR and was scientific advisor for Mydecine, Entheon Biomedical, Clerkenwell Health, Smallpharma Ltd. and Field Trip Health Ltd. SR received a salary award from the Fonds de recherche du Québec - Santé, receives grant funding from Mitacs, is on a steering committee for AbbVie, and owns shares of Aifred Health.

The remaining authors declare that the research was conducted in the absence of any commercial or financial relationships that could be construed as a potential conflict of interest.

## Publisher’s note

All claims expressed in this article are solely those of the authors and do not necessarily represent those of their affiliated organizations, or those of the publisher, the editors and the reviewers. Any product that may be evaluated in this article, or claim that may be made by its manufacturer, is not guaranteed or endorsed by the publisher.
